# Comparative clinical profiles and outcomes of prior vs. concurrently diagnosed atrial fibrillation in acute ischaemic stroke: the implication of diagnosis timing

**DOI:** 10.1093/europace/euaf107

**Published:** 2025-06-03

**Authors:** Hyo-Jeong Ahn, Young-Hae Go, So-Ryoung Lee, JungMin Choi, Kyung-Yeon Lee, Soonil Kwon, Eue-Keun Choi, Seil Oh, Gregory Y H Lip

**Affiliations:** Department of Internal Medicine, Seoul National University Hospital, 101 Daehak-ro, Jongno-gu, Seoul 03080, Republic of Korea; Department of Internal Medicine, Seoul National University Hospital, 101 Daehak-ro, Jongno-gu, Seoul 03080, Republic of Korea; Department of Internal Medicine, Seoul National University Hospital, 101 Daehak-ro, Jongno-gu, Seoul 03080, Republic of Korea; Department of Internal Medicine, Seoul National University College of Medicine, Seoul, Republic of Korea; Department of Internal Medicine, Seoul National University Hospital, 101 Daehak-ro, Jongno-gu, Seoul 03080, Republic of Korea; Department of Internal Medicine, Seoul National University Hospital, 101 Daehak-ro, Jongno-gu, Seoul 03080, Republic of Korea; Department of Internal Medicine, Seoul Metropolitan Government-Seoul National University Boramae Medical Center, Seoul, Republic of Korea; Department of Internal Medicine, Seoul National University Hospital, 101 Daehak-ro, Jongno-gu, Seoul 03080, Republic of Korea; Department of Internal Medicine, Seoul National University College of Medicine, Seoul, Republic of Korea; Department of Internal Medicine, Seoul National University Hospital, 101 Daehak-ro, Jongno-gu, Seoul 03080, Republic of Korea; Department of Internal Medicine, Seoul National University College of Medicine, Seoul, Republic of Korea; Department of Internal Medicine, Seoul National University College of Medicine, Seoul, Republic of Korea; Liverpool Centre for Cardiovascular Science at University of Liverpool, Liverpool John Moores University, Liverpool Chest and Heart Hospital, Liverpool, UK; Department of Clinical Medicine, Aalborg University, Aalborg, Denmark

**Keywords:** Atrial fibrillation, Stroke, Diagnosis timing

## Abstract

**Aims:**

Based on the diagnostic sequence in relation to stroke, a recent classification of atrial fibrillation (AF) categorizes AF into known AF (KAF) and AF detected after stroke or transient ischaemic attack (AFDAS). However, relatively little is known about AF ‘concurrently diagnosed with stroke’—perhaps the ‘grey zone’ of AF between KAF and AFDAS, which has been less characterized in terms of its resemblance to clinical characteristics and outcomes compared with AFDAS or KAF.

**Methods and results:**

Patients with AF who were admitted for acute ischaemic stroke (IS) in 2010–20 were retrospectively reviewed. Clinical characteristics and net clinical outcome (NCO)—the composite of recurrent stroke, major bleeding, hospitalization or emergency department visits for cardiovascular events, and death—were compared between AF diagnosed before stroke (prior AF) and incident AF diagnosed concurrently with IS (AFDCS). A total of 720 patients with AF and acute IS (mean age, 72.5 ± 10.1 years; 60.3% male) were included: prior AF, 62.6% (*n* = 451), and AFDCS, 37.4% (*n* = 269). Prior AF presented with more prevalent diabetes, heart failure, vascular disease, and valvular heart disease than AFDCS (all *P* < 0.05). The AFDCS had a significantly higher left ventricular ejection fraction and smaller left atrial diameter than prior AF. During a median follow-up of 2.0 (interquartile range 0.6–4.6) years, AFDCS was associated with a lower risk of NCO than prior AF without significant differences in the risk of recurrent stroke: adjusted hazard ratio (95% confidence interval), 0.776 (0.611–0.986), *P* = 0.038 for NCO and 0.784 (0.450–1.365), *P* = 0.389 for recurrent stroke.

**Conclusion:**

Prior AF and AFDCS have distinctive clinical profiles supporting AF is a disease of continuum according to its diagnostic vicinity to the IS. In terms of recurrent IS, AFDCS has a comparable risk with prior AF, indicating the importance of early detection and integrated management of AF for patients with IS.

## Graphical Abstract

**Graphical Abstract:**
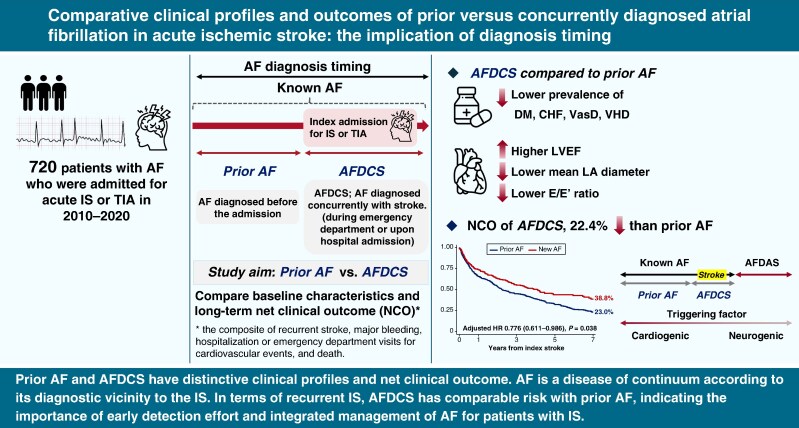

**What’s new?**

Atrial fibrillation (AF) can be classified based on the detection timing in relation to acute stroke. Atrial fibrillation diagnosed concurrently with stroke (AFDCS) presented a healthier vascular profile, less advanced atrial cardiomyopathy features, and a lower risk of net clinical outcome than prior AF (AF diagnosed before stroke), indicating the clinical spectrum of AF in relation to the diagnostic proximity to stroke. However, AFDCS presented a comparable risk of recurrent stroke with prior AF, which supports the prevailing notion that AFDCS is similar to known AF rather than AF diagnosed after stroke. Our finding supports that AF might be perceived as a continuum disorder depending on its detection in relation to stroke. Given the nature of AF—the more you look for AF, the more you find it—it is important to give active effort to diagnose AF during stroke admission, not only for elucidating the underlying stroke mechanism but also for guiding appropriate antithrombotic strategies and managing AF-related cardiovascular comorbidities.

## Introduction

Atrial fibrillation (AF) and ischaemic stroke (IS) are closely related to each other through their diagnostic time course.^1^ Atrial fibrillation is associated with a five-fold increased risk of IS,^2^ and around 40% of patients with IS or transient ischaemic attack (TIA) have AF.^1,3^ In detail, up to 30% of patients with stroke have known AF (KAF),^4,5^ and nearly a quarter of stroke patients who are believed to be AF-free are subsequently diagnosed with AF.^1,6^

In this context, a new classification of AF considering the diagnostic sequence in relation to stroke has been suggested.^7^ Based on different detection time frames, AF detected after stroke or TIA (AFDAS) has been proposed to differentiate patients with previous KAF before their stroke diagnosis.^7^ The patients with AFDAS may have distinctive clinical characteristics; for example, AFDAS has fewer cardiovascular (CV) comorbidities and AF-related cardiac substrate than KAF.^8^ Also, AFDAS is a different clinical entity from KAF, having a relatively more benign prognosis—with an overall lower risk of stroke—possibly explained by the mixture of both cardiogenic and neurogenic pathophysiology, whereas KAF predominantly rises from the cardiogenic factors.^9,10^

Meanwhile, there has been a shift in perceptions regarding AF diagnosed in the vicinity of the post-stroke period, whether it is detected in the emergency department (ED) or upon hospital admission.^7,11^ Initially, incident AF diagnosed near or shortly after stroke event was considered to be included in AFDAS^7^; however, it was reclassified as distinct from AFDAS and more closely to KAF since it may be a pre-existing arrhythmia and represent KAF, which was undiagnosed before presentation of the stroke.^11,12^ Such AF, so-called ‘AF concurrent diagnosis with stroke’, has been less characterized in terms of its resemblance to clinical characteristics and outcomes compared with AFDAS or KAF.

Hence, there is a need for a more in-depth examination of the clinical and prognostic characteristics of this ‘grey zone’ AF. This will offer a novel perspective on AF and redefine it as a more heterogeneous disease entity, considering its diagnostic sequence in relation to stroke.

From the cohort of patients with AF who were admitted for an acute IS, we aimed to investigate (i) baseline characteristics, (ii) prescription of antithrombotic therapy, and (iii) long-term outcomes according to the diagnostic sequence of AF and acute IS, namely by AF diagnosed before stroke (prior AF) vs. incident AF detected in the ED or upon hospital admission for stroke [i.e. AF diagnosed concurrently with stroke (AFDCS)].

## Methods

### Study population

From January 2010 to December 2020 at a tertiary referral hospital (Seoul National University Hospital), we retrospectively identified patients with prior or AFDCS who were admitted for IS or TIA with confirmation on brain magnetic resonance imaging (MRI) from their electrical medical records. A detailed description of the study population is described in our previous study evaluating post-stroke antithrombotic therapy in AF and their clinical prognosis.^13^ Among these, we excluded patients with a prior history of stroke and only included those with the first-ever episode of acute index stroke (IS).

The study population was classified into two groups based on their timing of AF diagnosis: (i) *prior AF* for those with AF diagnosed before the admission of acute IS and (ii) *AFDCS* for those with AF diagnosed on 12-lead electrocardiogram (ECG) or cardiac rhythm monitoring (telemonitor, 24 h-Holter, or extended ECG monitor patch) in the ED or upon hospital admission.

Three cardiologists (H.-J.A., S.-R.L., and Y.-H.G.) thoroughly reviewed each case and excluded misclassified patients who were subsequently diagnosed as AF after the index admission period or who were admitted for alternative neurological issues such as neuropathy or seizure. This study was approved by the institutional review board at Seoul National University Hospital (H-2105-165-1221). Written informed consent was waived, given all data were anonymized and de-identified.

### Covariates

Baseline clinical characteristics, including demographic information, anthropometric measurements, comorbidities, laboratory test findings, medication, smoking/drinking habits, and utilization of antithrombotic drugs, were investigated based on the index admission record. We obtained echocardiographic data nearest to the time of the index stroke, within a window of up to 6 months before or after the occurrence of the index stroke. The temporal association between the diagnosis of AF and acute IS was determined by reviewing past medical records, self-reported medical histories, and ECG recordings. Detailed information on stroke aetiology and MRI readings were also collected. Stroke aetiology was established based on the Trial of Org 10172 in Acute Stroke Treatment classification, with agreement reached among neurologists primarily involved in peri-stroke care; large artery atherosclerosis (LAA), cardioembolism (CE), small-vessel occlusion, stroke of other determined aetiology, and stroke of undetermined aetiology (UD).^13,14^ The severity of stroke was evaluated by the National Institutes of Health Stroke Scale (NIHSS) score based on the neurological evaluation records obtained at admission. National Institutes of Health Stroke Scale scores ≥5 and ≥15 were considered indicative of moderate or greater and severe stroke severity, respectively.^15^

### Study outcomes

The primary outcome was defined as the net clinical outcome (NCO), the composite of recurrent stroke, major bleeding, hospitalization or ED visits for CV events, and death. Major bleeding was defined as events meeting the criteria of the International Society on Thrombosis and Haemostasis, which is a reduction in the haemoglobin level of at least 20 g/L, transfusion of two or more units of blood, or symptomatic bleeding in a critical area or organ.^16^ The individual outcome was defined as a secondary outcome. The primary and secondary outcomes were assessed through participants’ self-reports during subsequent outpatient clinic visits, as well as through a consecutive review of healthcare utilization records at the study centre. National Statistical Information Service provided the death data of the included patients upon an official request. The outcomes were compared between prior AF and AFDCS from the onset of acute IS until either the occurrence of the outcomes or 31 December 2021, whichever came first, with the blanking period as the duration between the discharge date and the first clinic period.

### Statistical analysis

Continuous variables are presented as mean ± standard deviation or median [interquartile range (IQR)]. Student’s *t*-test or Mann–Whitney test was used to compare continuous variables between study groups. Categorical variables are described as numbers (%). Pearson’s *χ*^2^ or Fisher’s exact test was applied to compare categorical variables as required. We evaluated baseline clinical characteristics more associated with AFDCS than prior AF by a multivariable logistic regression model and reported as odds ratio (OR) with 95% confidence intervals (CIs).

A Cox proportional hazards regression model evaluated the risk of primary and secondary outcomes according to the timing of AF diagnosis (prior AF vs. AFDCS), and the comparison of the risk was presented as hazard ratio (HR) with 95% CI. Model 1 was unadjusted and Model 2 was adjusted by age and sex. Model 3 was adjusted by age, sex, diabetes mellitus, congestive heart failure, vascular disease, valvular heart disease, left ventricular ejection fraction (LVEF), left atrial (LA) diameter, and antithrombotic drug at the first clinic visit following discharge from the index admission, as these variables are representative distinctions between prior and AFDCS. Kaplan–Meier survival curve with a log rank or the Breslow test stratified and visualized the cumulative risks of the primary and secondary outcomes between prior AF and AFDCS. We performed a subgroup analysis comparing the risk of NCO between prior AF and AFDCS stratified by sex (male vs. female), age (age < 75 years vs. ≥75 years), and aetiology of stroke (non-CE vs. CE). All analyses were conducted using Stata (version 18, StataCorp LLC, College Station, TX, USA). Statistical significance was defined as a two-sided *P*-value of <0.05.

## Results

**Figure 1:**
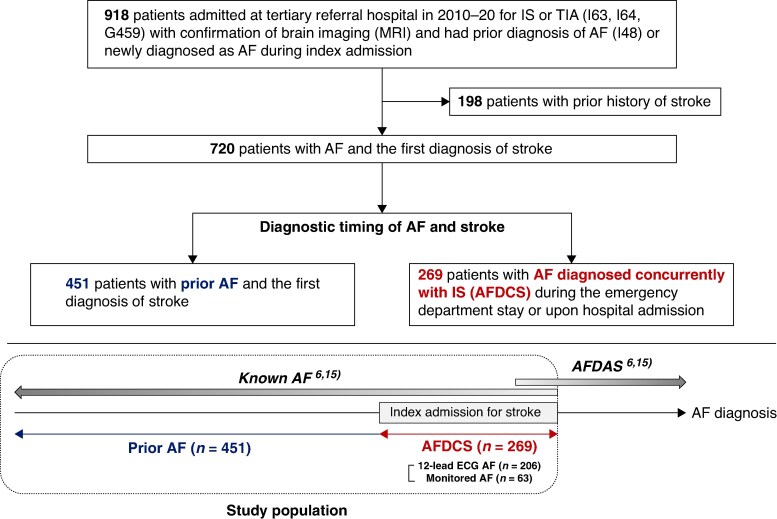
Inclusion of the study population. AF, atrial fibrillation; AFDAS, atrial fibrillation detected after stroke; AFDCS, atrial fibrillation diagnosed concurrently with stroke; ECG, electrocardiogram; IS, ischaemic stroke; MRI, magnetic resonance imaging; TIA, transient ischaemic attack.

We identified 918 patients admitted for IS or TIA with a prior history of AF or simultaneous diagnosis of AF and who had a brain MRI for the initial assessment between January 2010 and December 2020 from our priori study.^13^ Among them, 198 patients were excluded due to a prior history of stroke. Finally, 720 patients with AF experiencing their first diagnosis of stroke were included in the analysis of clinical characteristics. These patients were further categorized into two groups—prior AF vs. AFDCS—to evaluate clinical outcomes according to the diagnostic timing of AF and IS (*Figure 1*).

The baseline characteristics of the total population are shown in *Table 1*. The mean age was 72.5 ± 10.1 years, and 60.3% (*n* = 434) were male. The most common comorbidities were hypertension (62.9%) and diabetes mellitus (26.4%). The pre-stroke mean CHA_2_DS_2_-VASc score was 2.8 ± 1.4, and the mean HAS-BLED score was 2.3 ± 1.0. The LA of the overall population was dilated with a mean anteroposterior diameter of 49.4 ± 7.5 mm. The mean E/E′ ratio was elevated at 15.7 ± 14.5, suggesting the possibility of diastolic dysfunction. With respect to the aetiology of stroke, the most frequent cause was CE (72.4%), followed by UD (18.8%) and LAA (6.1%). The median duration of admission was 8.0 (5.0–15.0) days, and the median time from discharge to the first clinic visit was 20.0 (14.0–27.0) days.

**Table 1:** Baseline characteristics of the study population

	Total	Prior AF	AFDCS	*P*-value
	*N* = 720	*N* = 451 (62.6%)	*N* = 269 (37.4%)	
Age	72.5 ± 10.1	72.4 ± 10.2	72.7 ± 9.9	0.607
Male (%)	434 (60.3)	260 (57.6)	174 (64.7)	0.062
Body mass index (kg/m^2^)	24.0 ± 3.3	24.0 ± 3.2	23.9 ± 3.5	0.711
Comorbidities				
Hypertension (%)	453 (62.9)	282 (62.5)	171 (63.6)	0.781
Diabetes mellitus (%)	190 (26.4)	130 (28.8)	60 (22.3)	0.055
Congestive heart failure (%)	62 (8.6)	57 (12.6)	5 (1.9)	<0.001
Vascular disease (%)	124 (17.2)	91 (20.2)	33 (12.3)	0.007
Dyslipidaemia (%)	145 (20.1)	89 (19.7)	56 (20.8)	0.729
Valvular heart disease (%)	92 (12.8)	81 (18.0)	11 (4.1)	<0.001
CHA_2_DS_2_-VASc score	2.8 ± 1.4	3.0 ± 1.4	2.7 ± 1.4	0.007
HAS-BLED score	2.3 ± 1.0	2.3 ± 1.0	2.3 ± 1.0	0.599
Smoking (%)				0.015
Never smoker	446 (61.9)	295 (65.4)	151 (56.1)	
Ex-smoker	192 (26.7)	116 (25.7)	76 (28.3)	
Current smoker	81 (11.2)	40 (8.9)	41 (15.2)	
Current drinking (%)	241 (33.5)	131 (29.0)	110 (41.0)	<0.001
Medications (%)				
ACEi/ARB	171 (23.8)	129 (28.7)	42 (15.7)	<0.001
Beta-blockers	212 (29.5)	160 (35.6)	52 (19.4)	<0.001
Calcium channel blockers	136 (18.9)	103 (22.9)	33 (12.3)	<0.001
Diuretics	87 (12.1)	78 (17.3)	9 (3.4)	<0.001
Statin	145 (20.2)	98 (21.8)	47 (17.5)	0.168
Laboratory values				
White blood cell (10^3^/uL)	8.0 ± 2.9	7.9 ± 2.9	8.1 ± 2.9	0.281
Haemoglobin (g/dL)	13.5 ± 2.0	13.4 ± 2.0	13.7 ± 1.9	0.024
Haematocrit (%)	40.4 ± 5.5	40.2 ± 5.5	40.9 ± 5.4	0.065
BUN (mg/dL)	18.4 ± 8.6	18.6 ± 8.9	18.1 ± 8.1	0.523
Creatinine (mg/dL)	1.1 ± 1.0	1.2 ± 1.2	1.0 ± 0.6	0.123
eGFR (mL/min/1.73 m^2^)	72.5 ± 23.7	71.8 ± 25.0	73.6 ± 21.2	0.351
Total cholesterol (mg/dL)	161.7 ± 37.7	160.7 ± 37.2	163.5 ± 38.6	0.351
Triglyceride (mg/dL)	95.4 ± 43.5	94.8 ± 43.0	96.6 ± 44.4	0.612
HDL cholesterol (mg/dL)	47.8 ± 13.9	47.5 ± 14.4	48.4 ± 13.0	0.398
LDL cholesterol (mg/dL)	98.9 ± 33.6	97.8 ± 33.3	100.5 ± 33.9	0.313
Echocardiographic parameters (%)				
LVEF (%)	57.6 ± 8.7	56.7 ± 9.3	59.1 ± 7.4	<0.001
LVEF (≤40%)	40 (5.6)	31 (6.9)	9 (3.3)	0.041
LVEF (<50%)	80 (11.1)	61 (13.5)	19 (7.1)	0.006
LAD (mm)	49.4 ± 7.5	50.4 ± 7.5	47.9 ± 7.4	<0.001
LAD (≥50 mm)	335 (46.5)	227 (50.3)	108 (40.1)	0.001
E/E′	15.7 ± 14.5	16.7 ± 16.0	14.2 ± 11.5	0.032
E/E′ (≥15 mm)	235 (32.6)	162 (35.9)	73 (27.1)	0.009
AF information (%)				
Type				<0.001
Paroxysmal AF	393 (57.6)	182 (41.7)	211 (85.8)	
Non-paroxysmal AF	289 (42.4)	254 (58.3)	35 (14.2)	
CIED	—	24 (5.3)	2 (0.8)	0.002
Stroke information				
Time from AF diagnosis to incident stroke (days)	72.0 (0.0–1037.5)	623.0 (95.0–1693.0)	0.0 (0.0–0.0)	<0.001
Aetiology of stroke (TOAST classification) (%)				0.200
LAA	44 (6.1)	31 (6.9)	13 (4.8)	
CE	521 (72.4)	315 (69.8)	206 (76.6)	
SVO	17 (2.4)	13 (2.9)	4 (1.5)	
OD	3 (0.4)	3 (0.7)	0 (0.0)	
UD	135 (18.8)	89 (19.7)	46 (17.1)	
Microbleeds or haemorrhagic transformation on brain MRI (%)	268 (39.6)	169 (38.8)	99 (41.2)	0.531
NIHSS (%)	4.0 (2.0–12.0)	5.0 (2.0–12.0)	4.0 (2.0–12.0)	0.329
NIHSS ≥ 5	324 (45.0%)	210 (46.6%)	114 (42.4%)	0.265
NIHSS ≥ 16	121 (16.8%)	81 (18.0%)	40 (14.9%)	0.280
Duration of admission (days)	8.0 (5.0–15.0)	8.0 (5.0–16.0)	8.0 (6.0–15.0)	0.344
Time from discharge to the first visit (days)	20.0 (14.0–27.0)	20.0 (14.0–27.0)	19.0 (13.0–26.0)	0.013

Percentages may not total 100.0 because of rounding.

ACEi, angiotensin-converting enzyme inhibitors; AF, atrial fibrillation; AFDCS, atrial fibrillation diagnosed concurrently with ischaemic stroke; ARB, angiotensin II receptor blockers; BUN, Blood urea nitrogen; CE, cardioembolism; CIED, cardiac implantable electronic device; eGFR, estimated glomerular filtration rate; HDL, high-density lipoprotein; LAA, large artery atherosclerosis; LAD, left atrial diameter; LDL, low-density lipoprotein; LVEF, left ventricular ejection fraction; MRI, magnetic resonance imaging; OD, other determined aetiology; NIHSS, National Institutes of Health Stroke Scale; SVO, small vessel occlusion; TOAST, Trial of Org 10172 in Acute Stroke Treatment; UD, undetermined aetiology.

### Clinical characteristics of prior atrial fibrillation and atrial fibrillation diagnosed concurrently with ischaemic stroke

Of the 720 patients, 451 (62.6%) patients were diagnosed with AF before IS (prior AF) and 269 (37.3%) were diagnosed with AF simultaneously with IS (AFDCS). Differences in clinical characteristics between the two groups are shown in *Table 1*.

Compared with the prior AF, the AFDCS had a numerically higher proportion of males (57.6% vs. 64.7%, *P* = 0.062). Among comorbidities, the AFDCS had a lower prevalence of diabetes mellitus (28.8% vs. 22.3%, *P* = 0.055), congestive heart failure (12.6% vs. 1.9%, *P* < 0.001), vascular disease (20.2% vs. 12.3%, *P* = 0.007), and valvular heart disease (18.0% vs. 4.1%, *P* < 0.001). The pre-stroke mean CHA_2_DS_2_-VASc score was lower in the AFDCS than the prior AF (3.0 ± 1.4 vs. 2.7 ± 1.4, *P* = 0.007).

In terms of the type of AF, the prevalence of paroxysmal AF was higher in the AFDCS than the prior AF (41.7% vs. 85.8%, *P* < 0.001). Regarding echocardiographic parameters, the AFDCS had a higher mean LVEF (56.7 ± 9.3% vs. 59.1 ± 7.4%; the proportion of LVEF < 50%, 13.5% vs. 7.1%), lower mean LA diameter (50.4 ± 7.5 mm vs. 47.9 ± 7.4 mm; the proportion of LA diameter ≥ 50 mm, 50.3% vs. 40.1%), and lower E/E′ ratio (16.7 ± 16.0 vs. 14.2 ± 11.5; the proportion of E/E′ ≥ 15, 35.9% vs. 27.1%) than prior AF (all *P* < 0.05).

The method of AF detection and AF management within 3 years of AF diagnosis in AFDCS is summarized in Supplementary material online, *Table S1*. Most AFDCS cases were identified using 12-lead ECG (*n* = 206, 76.6%) or Holter monitoring (*n* = 40, 14.9%) during admission. The median AF burden was 30.0% (IQR, 9.0–100.0), and 40% (*n* = 16/40) had an AF burden ≥ 50%. The remaining cases were detected through telemonitoring, analysis of cardiac implantable electronic devices, or during other cardiac evaluations, such as transthoracic echocardiography. Approximately half of AFDCS (*n* = 137, 50.9%) was treated with anticoagulation alone without any rate or rhythm control therapy. Additional rate control was performed in 30.5% of patients (*n* = 82), while rhythm control was employed in only 18.6% (*n* = 50), predominantly using antiarrhythmic drugs. The brief comparison of the baseline characteristics within AFDCS based on AF detection method—12-lead ECG AF vs. the others (i.e. monitored AF)—is presented in Supplementary material online, *Table S2*.

In the multivariable logistic regression model (*Table 2*), clinical factors associated with the simultaneous diagnosis of AF (i.e. AFDCS) included a lower prevalence of diabetes mellitus [OR (95% CI), 0.655 (0.440–0.975); *P* = 0.037], congestive heart failure [OR (95% CI), 0.149 (0.052–0.427); *P* < 0.001], vascular disease [OR (95% CI), 0.616 (0.382–0.995); *P* = 0.048], and valvular heart disease [OR (95% CI), 0.334 (0.161–0.694); *P* = 0.003]. Additional clinical factors were a higher LVEF [OR (95% CI), 1.024 (1.002–1.047); *P* = 0.034] and a smaller LA diameter [OR (95% CI), 0.969 (0.947–1.010); *P* = 0.010].

**Table 2:** Multivariable logistic regression analysis of clinical factors associated with atrial fibrillation diagnosed concurrently with ischaemic stroke compared with prior atrial fibrillation

Clinical factors	Multivariable	
	OR (95% CI)	*P*-value
Male	0.839 (0.546–1.290)	0.425
Diabetes mellitus	0.655 (0.440–0.975)	0.037
Congestive heart failure	0.149 (0.052–0.427)	<0.001
Vascular disease	0.616 (0.382–0.995)	0.048
Valvular heart disease	0.334 (0.161–0.694)	0.003
Left ventricular ejection fraction (%)^a^	1.024 (1.002–1.047)	0.034
Left atrial diameter (mm)^a^	0.969 (0.947–0.993)	0.010
E/E′^a^	0.997 (0.984–1.010)	0.605
Smoking^b^	1.180 (0.882–1.578)	0.265
Current drinking	1.406 (0.936–2.111)	0.101

CI, confidence interval; OR, odds ratio.

^a^Odds ratios were calculated for each 1-unit increase in left ventricular ejection fraction, left atrial diameter, and E/E′.

^b^Smoking includes ex and current smoking, thus reflecting any lifetime exposure to smoking.

### Stroke site involvement, severity, and peri-stroke prescription pattern of antithrombotic therapy of prior atrial fibrillation and atrial fibrillation diagnosed concurrently with ischaemic stroke

The stroke site identified on brain MRI is described in Supplementary material online, *Table S3*. The most common stroke lesion was frontal lobe (45.6%) followed by parietal lobe (36.8%) and temporal lobe (36.1%). The proportion of AF patients who have insular cortex involvement as stroke site was 35.0% (*n* = 158) in prior AF and 37.2% (*n* = 100) in AFDCS (*P* = 0.210). The NIHSS score was numerically higher in prior AF than AFDCS, but without statistical significance: 5.0 (2.0–12.0) in prior AF and 4.0 (2.0–12.0) in AFDCS, *P* = 0.329.

The prescription pattern of antithrombotic drugs at four distinct time points is summarized in Supplementary material online, *Figure S1*. Prior to the occurrence of the index stroke, 28.4% of the prior AF group and 72.9% of the AFDCS group were not utilizing oral anticoagulants (OAC) or antiplatelets (APT). Among 245 prior AF patients who were admitted since 2015, when direct oral anticoagulant (DOAC) began to be actively prescribed in Korea, 202 patients had CHA_2_DS_2_-VASc Score ≥ 2 thus indicated for OAC. However, 117 (57.9%) patients were not using OAC; instead, 56/117 patients (47.9%) were using APTs.

During the admission period of index IS, 97.8% of the prior AF group received a prescription for either OAC or APT(s) with 37.3% for exclusive OAC prescription. In the AFDCS group, 98.8% received a prescription for either OAC or APT(s) with 27.1% for exclusive OAC prescription. Compared with the prior AF, the proportion of patients prescribed both OAC and APT(s) was higher in AFDCS (47.9% vs. 62.8%). In total, 13.1% (94/720), 11.4% (73/642), and 10.3% (54/522) were not treated with OAC during stroke admission, at the first clinic after discharge, and 1 year after stroke, respectively.

At the first clinic visit after discharge, data for 89.2% (*n* = 642) of the total population were accessible. Among prior AF, 63.4% were prescribed OAC alone, 10.6% were taking APT(s) alone, and 24.0% patients were on both OAC and APT(s). In the AFDCS group, 73.2% were prescribed OAC alone, 6.9% were taking APT(s) alone, and 17.5% were on both OAC and APT(s). There was no significant difference in the prescription regimens of OAC and APT(s) between the two groups. The distribution of the antithrombotic therapy at 1 year after the index IS was similar to that of the first clinic after discharge, and there was no significant difference between the two groups.

### Clinical outcomes of prior atrial fibrillation vs. atrial fibrillation diagnosed concurrently with ischaemic stroke

During a median follow-up of 2.0 (0.6–4.6) years, the overall incidence rate per 100 patient-year was 21.72 for NCO; 2.84 for recurrent stroke; 1.88 for major bleeding; 13.49 for hospitalization or ED visits for CV events; and 8.35 for death. The proportion of the transition from paroxysmal to persistent AF during follow-up was 31.9% (*n* = 58) in prior AF and 30.3% (*n* = 64) in AFDCS (*P* = 0.577).

**Figure 2:**
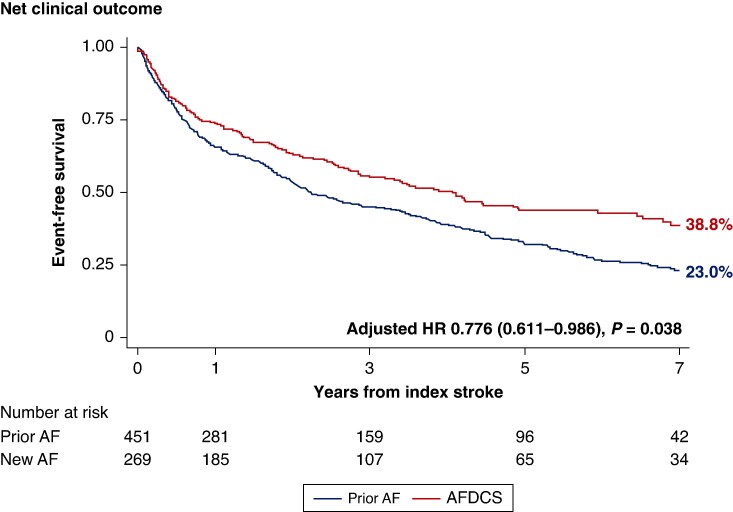
Cumulative risk of net clinical outcome in patients with atrial fibrillation and stroke after discharge from the index stroke stratified by the diagnostic timing of atrial fibrillation and stroke. Hazard ratios were adjusted by age, sex, diabetes mellitus, congestive heart failure, vascular disease, valvular heart disease, left ventricular ejection fraction, left atrial diameter, and antithrombotic therapy at the first clinic visit following discharge from the index admission. AF, atrial fibrillation; AFDCS, atrial fibrillation diagnosed concurrently with stroke; HR, hazard ratio.

The cumulative risk of the primary outcome, NCO, according to the diagnostic timing of AF and stroke is presented in *Figure 2* and Supplementary material online, *Table S4*. At 7.0 years from the occurrence of index IS, compared with the prior AF, the AFDCS group showed a significantly lower risk of NCO (adjusted HR 0.776, 95% CI 0.611–0.986, *P* = 0.038).

**Figure 3:**
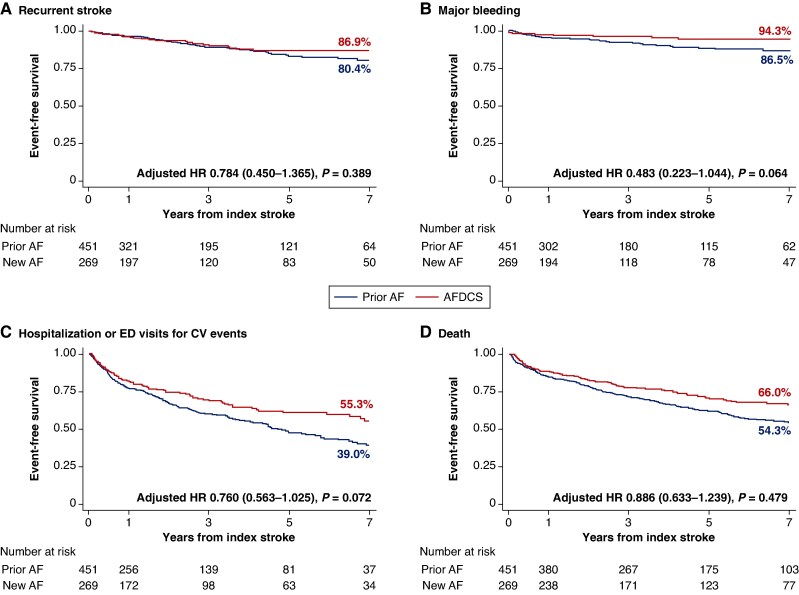
Cumulative risks of (*A*) recurrent stroke, (*B*) major bleeding, (*C*) hospitalization or emergency department visits for cardiovascular events, and (*D*) death in patients with atrial fibrillation and stroke after discharge from the index stroke stratified by the diagnostic timing of atrial fibrillation and stroke. Hazard ratios were adjusted by age, sex, diabetes mellitus, congestive heart failure, vascular disease, valvular heart disease, left ventricular ejection fraction, left atrial diameter, and antithrombotic therapy at the first clinic visit following discharge from the index admission. AF, atrial fibrillation; AFDCS, atrial fibrillation diagnosed concurrently with stroke; CV, cardiovascular; ED, emergency department; HR, hazard ratio.

For secondary outcomes, the AFDCS group showed marginally lower risk of major bleeding (adjusted HR 0.483, 95% CI 0.223–1.044, *P* = 0.064) and lower risk of hospitalization or ED visits for CV events (adjusted HR 0.760, 95% CI 0.563–1.025, *P* = 0.072) than the prior AF group. There was no significant difference in recurrent stroke between the two groups (log-rank *P* = 0.323). The risk of death was progressively lower in AFDCS than prior AF, but this was not statistically significant after multivariable adjustments (adjusted HR 0.886, 95% CI 0.633–1.239, *P* = 0.479) (*Figure 3*; Supplementary material online, *Table S3*).

The cumulative risk of NCO within AFDCS discriminated by AF detection method (12-lead ECG AF vs. monitored AF) is presented in Supplementary material online, *Figure S2*. The monitored AF showed a lower trend of NCO than 12-lead ECG AF but was not significantly different: adjusted HR (95% CI), 0.647 (0.405–1.035), *P* = 0.070.

### Subgroup analyses

Subgroup analyses were performed for the comparison of NCO between prior AF and AFDCS. For NCO, the lower risk in AFDCS than prior AF was greater in males than females (*P* for interaction = 0.004) (see Supplementary material online, *Figure S3*). The lower risk of NCO in AFDCS than prior AF was consistent irrespective of age (age < 75 years vs. ≥75 years) and aetiology of stroke (non-CE vs. CE).

## Discussion

From this single-centre retrospective cohort of patients with AF who admitted for their first-ever episode of acute IS, our principal findings are as follows: (i) approximately one-third of AF-acute IS patients are newly diagnosed with AF during their ED stay or admission for acute IS; (ii) patients with AFDCS had fewer comorbidities, higher LVEF, and smaller LA than prior AF; (iii) there were no differences in the utilization of antithrombotic drugs between prior AF and AFDCS following discharge from the index stroke admission; (iv) the risk of NCO was 22.4% lower in the AFDCS than prior AF group, particularly amongst male patients; and (v) the risk of recurrent stroke was not significantly different between prior AF and AFDCS.

The improvement in prolonged cardiac monitoring technologies has led to the development of a novel approach to understanding AF, categorizing it according to its diagnostic timing in relation to IS.^17^ The classification of AF as KAF and AFDAS was first proposed in 2017,^7^ and distinctive clinical profiles were acknowledged.^8^ More explicitly, AFDAS was defined as AF detected on short-term (e.g. 24- or 48-h Holter) or prolonged cardiac monitoring (e.g. ≥7 days) after acute IS or TIA in patients without a history of AF.^18^ However, the patient profile of AF detected during ED or hospital admission has been less demonstrated, and there has been a shift in how this ‘simultaneously diagnosed AF’ is perceived. Contrary to the initial consideration of this simultaneous AF as AFDAS, later it was regarded as KAF as it is more likely to be a pre-existing arrhythmia and reflect a high burden or persistent AF.^11^ Nonetheless, there is limited evidence about whether this so-called AF diagnosed during acute illness should be categorized as KAF in terms of clinical characterization and evaluation of prognosis,^19,20^ especially in Asian patients.

In the present study, we identify AFDCS—incident AF detected in the ED or upon hospital admission for stroke—as have distinctive clinical profiles than prior AF, although both are often regarded under the same category of KAF. The AFDCS group has less comorbidities, higher LVEF, and smaller LA than prior AF. From that, we acknowledged that a spectrum of CV health is observed even within KAF according to their diagnostic proximity to acute IS.

Interestingly, we found clinical prognosis can be differentiated between prior AF and AFDCS. To the best of our knowledge, this is the first report of the composite risk of clinical events—recurrent stroke, bleeding, hospitalization or ED visits, or death—according to the diagnostic timing of AF and acute IS. The lower risk of NCO in AFDCS than prior AF consistently remained after multiple adjustments, including exposure to antithrombotic use after index stroke. It appears evident that AFDCS presents with a more favourable prognosis than prior AF, characterized by a healthier vascular profile and less cardiac remodelling on imaging. The overall more favourable prognosis observed in patients with AFDCS compared with those with prior AF may be interpreted in the context of atrial cardiomyopathy progression. Specifically, AFDCS may reflect a less advanced stage of atrial cardiomyopathy, as suggested by a smaller LA diameter and fewer associated comorbidities.^21^ Our finding supports that AF might be perceived as a continuum disorder, particularly in terms of the grade of atrial cardiomyopathy progression, with varying future clinical risks depending on when it is detected in relation to acute IS, which extends the current classification of AF.

Nonetheless, it is pertinent to highlight that despite the healthier vascular profiles and less advanced cardiac remodelling observed in cases of AFDCS compared with prior AF, there were no discernible differences in the risk of recurrent stroke. Our finding aligns with a recent retrospective observational cohort study of IS/TIA patients with AF, which reported no significant difference in the risk of recurrent IS between AF known before stroke occurrence (referred to here as prior AF) and newly diagnosed AF on a 12-lead ECG during the initial assessment or stroke admission (referred to here as AFDCS).^22^ Regarding the risk of recurrent stroke, the Ontario Stroke Registry data reported that AFDAS has a lower risk of recurrent stroke than KAF.^5^ In this study, we observed that despite the overall better clinical prognosis, AFDCS has a similar risk of recurrent stroke with prior AF. This finding supports the prevailing consensus that AFDCS is more likely to be classified as KAF than AFDAS.^11,18^ In other words, our data reinforce the idea that AFDCS, in conjunction with prior AF, should be classified under the same category of KAF in terms of their association with recurrent stroke risk. Indeed, our available Holter monitoring data in AFDCS suggest that a considerable AF burden may be inherently present in the AFDCS group as presumed to be in prior AF, potentially contributing to the index stroke event and further contributing to the comparable subsequent stroke risk. Meanwhile, in the subgroup analysis of AFDCS by AF detection method, monitored AF showed a trend towards lower NCO risk and smaller LA, implying a less advanced form of AF when detected prospectively and further from the stroke event. This is in line with the report from London Ontario Stroke Registry—AF detected on 12-lead ECG during the ED or stroke has five-fold higher recurrent IS risk than prolonged cardiac monitoring detected AF after stroke^23^—that should be validated in a larger cohort with stroke-AF detection time-varying analysis.

Collectively, the results regarding NCO and recurrent stroke risk indicate that AF is a disease of continuum depending on the stroke time relation, although AFDCS (i.e. ECG-AF) should be still categorized under a KAF in terms of IS recurrence rate.

The distinctive pathophysiology of AF—the interplay and mixture of cardiogenic and neurogenic forces—may account for the varying clinical outcomes.^9,10,24^ Conceptually, AFDCS might have more neurogenic and less cardiogenic factors than prior AF. Indeed, AFDCS could arise from the autonomic and inflammatory regulation between the brain and heart as in the context that AFDAS has been recognized as part of the recently described stroke-heart syndrome.^10,25^ Accordingly, involvement of the insular cortex, a trigger for neurogenic cardiac changes, is more frequent in AFDAS than KAF and suggests AF detected near acute IS (i.e. AFDCS) may have more neurogenic forces than AF detected apart from acute IS (i.e. prior AF).^26,27^ In our study, we observed a numerically higher stroke involvement of insular cortex in AFDCS than prior AF supporting a hypothesis that AF detected near the time of stroke might have influenced by stronger neurogenic forces. The potential predominance of neurogenic mechanisms and less severe atrial cardiopathy in AFDCS would be expected to have better clinical outcomes than KAF. The concept of perceiving AF considering the detection timing with acute IS that has been less frequently investigated thus far would provide us a better understanding and management strategy of AF, integrating additional elements beyond classical CV risk factors.^5,28,29^

However, although we observed AFDCS is associated with a better clinical prognosis than prior AF, OAC waive among these population should be cautiously approached since AFDCS presented a comparable recurrent IS risk with prior AF and AF-related stroke is more frequently fatal or disabling than in non-AF patients.^6,30^ Indeed, we noted OAC was undertreated in a considerable portion of AF-IS patients before and even after IS event. The 57.9% underutilization of OAC in prior AF of our cohort is consistent with the recent announced AF factsheet in Korea 2024. Although overall OAC utilization rate has improved since the DOAC introduction from 2013 to 2022, the proportion of patients with AF requiring OAC use, but not prescribed OACs within 6 months of AF diagnosis, was 51% in the pooled cohort between 2013 and 2022.^31^ The OAC underuse after IS—probably assumed due to the old age, concurrent cerebral microbleeding issues, or not primarily managed by cardiologists—is also reported in other nations cohort.^32,33^ Although diagnostic timing of AF in relation to IS may diversify individual AF profile, physicians should ensure adherence to a comprehensive approach to AF management once AF is detected.^34^ Moreover, given the nature of AF—the more you look for AF, the more you find it^35–37^—we believe that the clinical implication of our findings also lies in highlighting the importance of active efforts to diagnose AF during stroke admission. Such efforts are essential not only for elucidating the underlying stroke mechanism but also for guiding appropriate antithrombotic strategies and managing AF-related CV comorbidities.

### Limitations

There are several limitations in this study. First, our study may not fully represent AF and acute IS patients since the observational cohort was retrospectively established at a single tertiary referral centre. Second, clinical outcomes might be under-detected as the collection was based on the participants’ self-reports or administrative or outpatient clinic records exclusively from our centre. The absence of systematic prospective follow-up, combined with unmeasured confounding, could introduce a bias in assessing the risk of clinical outcomes based on the timing of AF diagnosis and acute IS. Third, we lacked information on the burden of AF, which could provide additional characterization of both prior and AFDCS. Lastly, the considerable undertreatment with OAC, which was observed in both groups, may have influenced the risk of clinical outcomes, particularly the risk of recurrent stroke. This treatment gap may limit the interpretation of the comparative risks associated with the timing of AF diagnosis. Nevertheless, our study suggests clinical discrimination and supports a new perspective of AF considering the temporal relationship with the diagnosis of acute IS from a large cohort with sufficient clinical data.

## Conclusion

Atrial fibrillation could be classified based on the detection timing frame in relation to acute IS. Both prior AF and AFDCS are under the same category of KAF. However, our findings highlight a clinical spectrum between prior and AFDCS, characterized by differences in comorbidity profiles and the prognosis of NCO, supporting the concept that AF is a disease of a continuum. We further reinforced the prevailing notion that AFDCS (AF detected simultaneously with stroke, or AF detected on 12-lead ECG during the initial assessment of stroke, referred to as ECG-AF) is similar to KAF than AFDAS in the aspect of comparable recurrent IS risk with prior AF. Diverse AF pathophysiology including neurogenic and cardiogenic triggers may contribute to the development of ‘grey zone’ AF, specifically AFDCS, in its temporal association with IS. Although there is a differentiation in clinical prognosis in accordance with the diagnostic vicinity to IS, multidimensional management strategy and effort to detect AF should be warranted as AF accounts for up to one-third of all strokes, and AF-related IS is more fatal or disabling than non-AF patients.

## Supplementary Material

euaf107_Supplementary_Data

## Data Availability

All data generated or analysed during this study are included in this published article and its Supplementary material. The datasets used and analysed during the current study are available from the corresponding author on reasonable request.
