# Supplementary material for: Comparative clinical profiles and outcomes of prior vs. concurrently diagnosed atrial fibrillation in acute ischaemic stroke: the implication of diagnosis timing
Source: Europace. 2025 Jun 3;27(6):euaf107. doi: 10.1093/europace/euaf107 (PMC12166361; doi:10.1093/europace/euaf107)
Supplement: euaf107_Supplementary_Data [file euaf107_supplementary_data.docx]

**Supplementary materials**

**Supplementary Table 1.** The detection and management of atrial fibrillation in atrial fibrillation diagnosed concurrently with stroke group.

**Supplementary Table 2.** The brief baseline characteristics of 12-lead ECG detected atrial fibrillation and monitored atrial fibrillation.

**Supplementary Table 3.** The stroke site of involvement according to the diagnostic timing of atrial fibrillation and stroke.

**Supplementary Table 4.** The risk of clinical outcomes in patients with atrial fibrillation and stroke after discharge from the index stroke according to the diagnostic timing of atrial fibrillation and stroke.

**Supplementary Figure 1.** Peri-stroke prescription pattern of antithrombotic therapy.

**Supplementary Figure 2**. The cumulative risk of the net clinical outcome according to the detection method of atrial fibrillation in atrial fibrillation diagnosed concurrently with stroke group.

**Supplementary Figure 3.** Subgroup analysis of net clinical outcome according to the diagnostic timing of atrial fibrillation and stroke.

**Supplementary Table 1.** The detection and management of atrial fibrillation in new atrial fibrillation group.

| Method of AF detection | | Management of AF | |
| --- | --- | --- | --- |
| 12-lead ECG | 206 (76.6%) | Anticoagulation alone | 137 (50.9%) |
| Holter monitoring | 40 (14.9%) | Anticoagulation + Rate control | 82 (30.5%) |
| Telemonitoring during admission  14 (5.2%) | | Anticoagulation + Rhythm control ^b^ | 50 (18.6%) |
|  |  | Antiarrhythmic drug | 44 (16.4%) |
| Others^a^ | 9 (3.3%) | DC cardioversion | 1 (0.4%) |
|  | | Catheter ablation | 9 (3.3%) |
|  |  | Open or thoracoscopic MAZE op. | 7 (2.6%) |

^a^ Others: AF detected on cardiac implantable electronic devices or during cardiac evaluation (i.e., echocardiography)

^b^ Multiple modalities of rhythm control were allowed.

**Supplementary Table 2.** The brief baseline characteristics of 12-lead ECG detected atrial fibrillation and monitored atrial fibrillation.

|  | AF diagnosed concurrently with stroke (AFDCS) | | p-value |
| --- | --- | --- | --- |
|  | 12-lead ECG AF (n=206) | Monitored AF  (n=63) |  |
| Age | 72.9±9.5 | 72.3±11.3 | 0.712 |
| Male | 132 (64.1%) | 42 (66.7%) | 0.709 |
| CHA_2_DS_2_-VASc score | 2.6±1.4 | 2.7±1.5 | 0.861 |
| HAS-BLED score | 2.3±1.0 | 2.1±1.1 | 0.245 |
| LAD (mm) | 48.5±7.1 | 45.8±8.1 | 0.010 |
| NIHSS | 4.0 (2.0-12.0) | 3.0 (2.0-11.0) | 0.422 |
| AF burden | - | 30.0 (9.0-100.0) % from available Holter data | - |

Abbreviations: AF, atrial fibrillation; LAD, left atrial diameter; NIHSS, National Institutes of Health Stroke Scale.

**Supplementary Table 3.** The stroke site of involvement according to the diagnostic timing of atrial fibrillation and stroke.

| Site of involvement | Total (n=720) | Prior AF (n=451) | New AF (n=269) |
| --- | --- | --- | --- |
| Frontal lobe | 328 (45.6%) | 207 (45.9%) | 121 (45.0%) |
| Parietal lobe | 265 (36.8%) | 156 (34.6%) | 109 (40.5%) |
| Temporal lobe | 260 (36.1%) | 152 (33.7%) | 108 (40.1%) |
| Occipital lobe | 125 (17.4%) | 76 (16.9%) | 49 (18.2%) |
| Deep subcortical structures | 236 (32.8%) | 140 (31.0%) | 96 (35.7%) |
| Cerebellum | 92 (12.8%) | 54 (12.0%) | 38 (14.1%) |
| Brainstem | 54 (7.5%) | 38 (8.4%) | 16 (5.9%) |
| Insular cortex | 258 (35.8%) | 158 (35.0%) | 100 (37.2%) |

* Total percentages may exceed 100% due to the presence of multiple stroke sites in some patients.

**Supplementary Table 4.** The risk of clinical outcomes in patients with atrial fibrillation and stroke after discharge from the index stroke according to the diagnostic timing of atrial fibrillation and stroke.

| **Diagnostic timing of AF and stroke** | **Event / N** | **Cumulative incidence** | **Incidence rate (100PY)** | **HR (95% CI)** | | |
| --- | --- | --- | --- | --- | --- | --- |
|  |  |  |  | **Model 1** | **Model 2** | **Model 3** |
| **Net clinical outcome** | | | | | | |
| Prior AF | 297/451 | 77.0% | 24.87 | 1 (Reference) | 1 (Reference) | 1 (Reference) |
| AFDCS | 135/269 | 61.2% | 17.05 | 0.700 (0.571-0.858) | 0.678 (0.552-0.832) | 0.776 (0.611-0.986) |
|  |  |  |  | p=0.001 | p<0.001 | p=0.038 |
| **Recurrent stroke** | | | | | | |
| Prior AF | 46/451 | 19.7% | 3.12 | 1 (Reference) | 1 (Reference) | 1 (Reference) |
| AFDCS | 22/269 | 13.1% | 2.40 | 0.775 (0.466-1.288) | 0.758 (0.455-1.262) | 0.784 (0.450-1.365) |
|  |  |  |  | p=0.325 | p=0.287 | p=0.389 |
| **Major bleeding** | | | | | | |
| Prior AF | 35/451 | 13.5% | 2.43 | 1 (Reference) | 1 (Reference) | 1 (Reference) |
| AFDCS | 10/269 | 5.7% | 1.02 | 0.439 (0.218-0.884) | 0.428 (0.212-0.862) | 0.483 (0.223-1.044) |
|  | | | | p=0.021 | p=0.018 | p=0.064 |
| **Hospitalization or ED visits for CV events** | | | | | | |
| Prior AF | 168/451 | 61.0% | 15.69 | 1 (Reference) | 1 (Reference) | 1 (Reference) |
| AFDCS | 77/269 | 44.8% | 10.36 | 0.693 (0.529-0.909) | 0.693 (0.528-0.909) | 0.760 (0.563-1.025) |
|  |  |  |  | p=0.008 | p=0.008 | p=0.072 |
| **All-cause death** |  |  |  |  |  |  |
| Prior AF | 174/451 | 45.7% | 9.57 | 1 (Reference) | 1 (Reference) | 1 (Reference) |
| AFDCS | 78/269 | 34.0% | 6.51 | 0.702 (0.538-0.917) | 0.685 (0.524-0.897) | 0.886 (0.633-1.239) |
|  |  |  |  | p=0.009 | p=0.006 | p=0.479 |

Abbreviation: AF, atrial fibrillation; AFDCS, atrial fibrillation diagnosed concurrently with stroke; CI, confidence interval; CV, cardiovascular; ED, emergency department; HR, hazard ratio; PY, person-year

Model 1: unadjusted.

Model 2: adjusted by age and sex.

Model 3: adjusted by age, sex, diabetes mellitus, congestive heart failure, vascular disease, valvular heart disease, left ventricular ejection fraction, left atrial diameter, and antithrombotic therapy at the first clinic visit following discharge from the index admission.

* 7-year event, cumulative incidence, and incidence rate

**Supplementary Figure 1.** Peri-stroke prescription pattern of antithrombotic therapy.


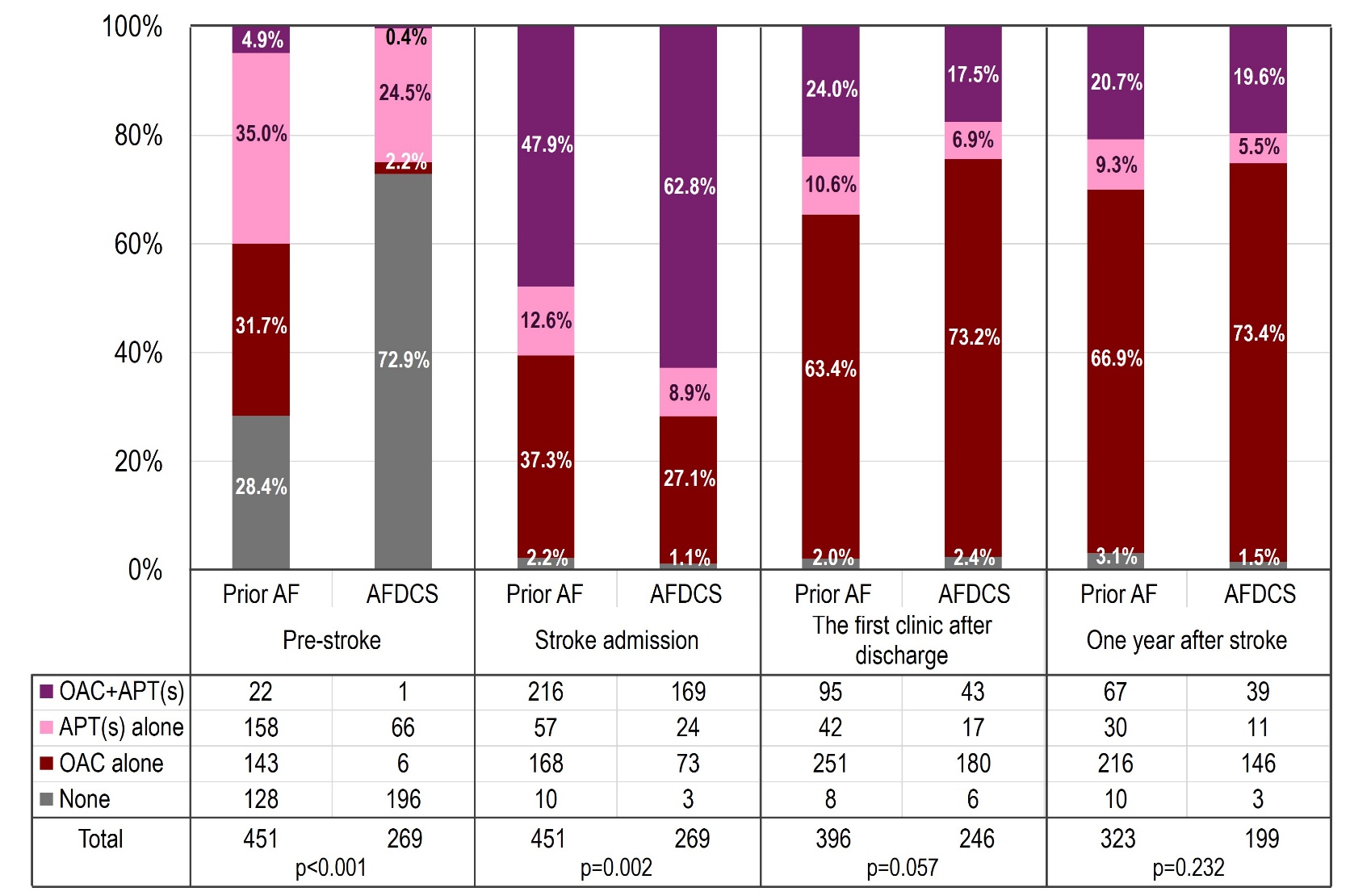


Abbreviations: AF, atrial fibrillation; AFDCS, atrial fibrillation diagnosed concurrently with stroke; APT, antiplatelet; OAC, oral anticoagulant.

Percentages may not total 100.0 because of rounding.

**Supplementary Figure 2**. The cumulative risk of the net clinical outcome according to the detection method of atrial fibrillation in atrial fibrillation diagnosed concurrently with stroke group.


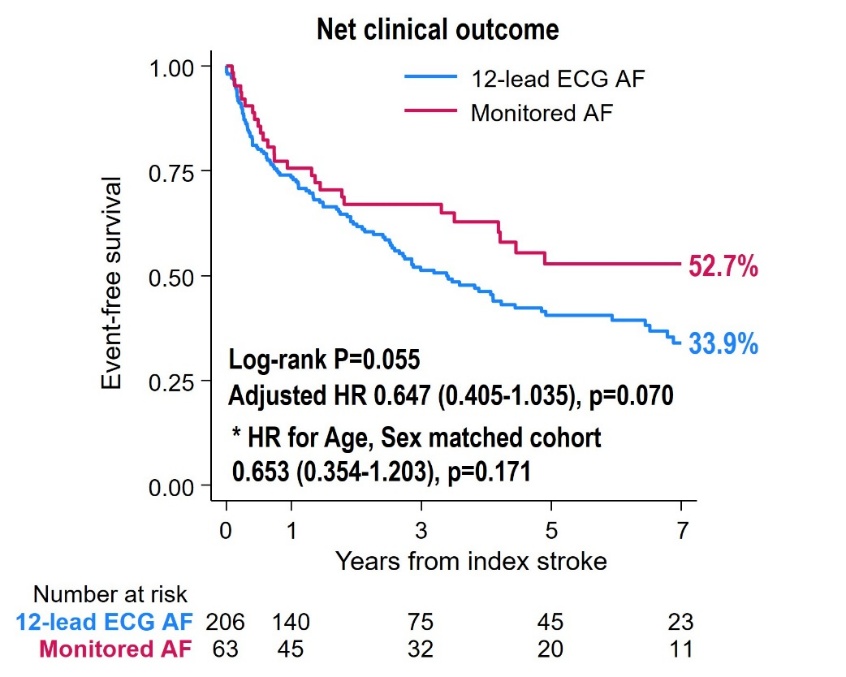


Abbreviations: AF, atrial fibrillation; HR, hazard ratio.

**Supplementary Figure 3.** Subgroup analysis of net clinical outcome according to the diagnostic timing of atrial fibrillation and stroke.


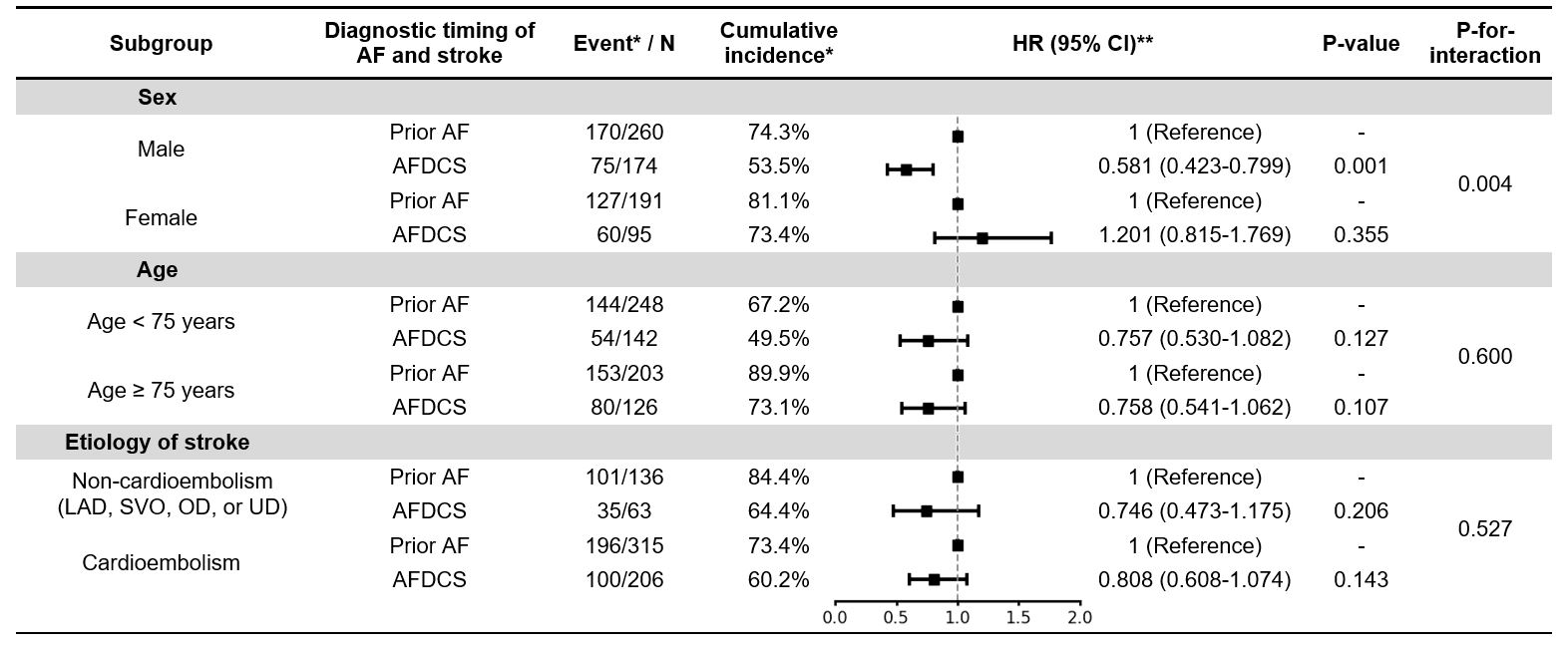


Abbreviation: AF, atrial fibrillation; AFDCS, atrial fibrillation diagnosed concurrently with stroke; CI, confidence interval; HR, hazard ratio; LAD, large artery disease; OD, other determined etiology; SVD, small vessel occlusion; UD, undetermined etiology.

* 7-year event, cumulative incidence, and incidence rate

** Adjusted by age, sex, diabetes mellitus, congestive heart failure, vascular disease, valvular heart disease, left ventricular ejection fraction, left atrial diameter, and antithrombotic therapy at the first clinic visit following discharge from the index admission.
